# Response to “Comment on ‘Fluorotechnology Is Critical to Modern Life: The FluoroCouncil Counterpoint to the Madrid Statement’”

**DOI:** 10.1289/ehp.1510295

**Published:** 2015-07-01

**Authors:** Jessica S. Bowman

**Affiliations:** FluoroCouncil

The FluoroCouncil’s voluntary development of alternative chemistries is unprecedented and a model for the development and introduction of more sustainable chemistry worldwide. More than a decade ago, the FluoroCouncil member companies responded to concerns about long-chain poly- and perfluoroalkyl substances (PFASs) by working with regulators to voluntarily phase out those substances and develop alternatives with improved health and environmental profiles. The FluoroCouncil also works with regulators and other stakeholders to support a global transition away from long-chain PFASs. This effort stands as a historic collaboration by government and industry to foster sustainable development.

The claim that all PFASs are problematic is simply not supported by the wealth of data available on both long- and short-chain PFASs. Because of the concerns raised in regard to long-chain PFASs, the U.S. Environmental Protection Agency (EPA) has held the fluorotechnology industry to high standards and increased data requirements to ensure the alternatives are well studied and safer than the substances being replaced. Consequently, short-chain PFASs are some of the most robustly studied new chemicals introduced to the market, having undergone years of toxicity and environmental testing at the request of regulators. Industry continues this collaboration with regulators, developing additional data on the alternatives and working to make those data publicly available, including on the FluoroCouncil website (http://www.fluorocouncil.org/Resources/Research). Based on this robust body of data, regulators globally have determined the alternatives are safe for their intended use. The “sustainable and less hazardous alternatives” sought by the Madrid Statement authors already exist in the form of short-chain PFASs.

We continue to be perplexed by assertions from the authors of the Madrid Statement that short-chain PFASs present hazards comparable to those of long-chain PFASs, citing publications such as Bull et al. (2014) and Wang et al. (2015). These publications suffer from important data gaps, such as the failure to cite key published articles on the toxicity of short-chain PFASs (e.g., Klaunig et al. [2015], which presents animal data indicating perfluorohexanoic acid is not carcinogenic). Furthermore, these publications actually acknowledge or demonstrate that many of the leading short-chain PFASs are less bioaccumulative and less toxic than the long-chain PFASs with which they have been compared, based on the available data taken as a whole. This conclusion, which is well accepted by regulatory agencies such as the EPA, compels a different policy outcome than the Madrid Statement suggests. The first priority for risk management should be phase-out of the long-chain PFASs. Attempting to broaden that phase-out to effective alternatives that are less hazardous can only create a technological impasse that supports the retention of long-chain PFASs in the marketplace.

The authors of the Madrid Statement also contend that PFASs are not critical to modern life. The importance of PFAS chemistry, however, was long ago determined by the market. Industries relying on PFASs evaluated fluorinated and nonfluorinated alternatives, as well as alternative technology, and decided on the products that met their specifications and performance needs. Some decisions involved continuing to use PFASs because they meet performance needs that nonfluorinated alternatives cannot. For example, first-responder protective gear is treated with fluorinated products to help maintain performance in fires; firefighting foam produced with fluorinated surfactants provides shorter extinguishment times and critical burnback resistance when fighting flammable liquid fires; and hospital gowns, drapes, and divider curtains rely on fluorinated polymers to provide protective barriers against transmission of diseases. Because the short-chain PFASs have been reviewed and approved by regulatory authorities globally, all applications relying on these substances can be used without presenting a significant risk.

The largest use of short-chain PFASs is for polymeric products. These products—like other polymers—are quite stable under environmental conditions. The resilience of short-chain PFASs is directly connected to its performance, providing long-lasting, durable properties. First responders, medical personnel, and patients would certainly not want the properties in safety gear or medical garments to quickly become ineffective. Even when the short-chain PFASs, which do not present a significant risk, are used in what some people may characterize as “nonessential applications,” such as clothing and furniture, these substances significantly extend the effective lifetime of those products, meaning less waste, infrequent washings, and economic savings. These benefits of short-chain PFASs can be further enhanced by reducing emissions through the adoption of best environmental practices, which the FluoroCouncil has identified and is encouraging in the supply chain.

The FluoroCouncil members remain committed to science-based stewardship activities, including continually enhancing the sustainability of their chemistries and products by improving their environmental, health, safety, and performance profiles. We are open to working collaboratively and constructively with stakeholders on *1*) strategies to complete the global transition away from long-chain PFASs, *2*) identification of issues that warrant further data development and risk assessment, *3*) actions that can foster additional stewardship activities within the supply chain, and *4*) best methods for transparently sharing information relevant to the health and environmental impact of PFASs.
